# Beyond Self-Recycling: Cell-Specific Role of Autophagy in Atherosclerosis

**DOI:** 10.3390/cells10030625

**Published:** 2021-03-11

**Authors:** James M. Henderson, Christian Weber, Donato Santovito

**Affiliations:** 1 Institute for Cardiovascular Prevention (IPEK), Ludwig-Maximillians-Universität (LMU), D-80336 Munich, Germany; James.Henderson@med.uni-muenchen.de; 2 German Center for Cardiovascular Research (DZHK), Partner Site Munich Heart Alliance, D-80336 Munich, Germany; 3 Department of Biochemistry, Cardiovascular Research Institute Maastricht (CARIM), Maastricht University, 6229 ER Maastricht, The Netherlands; 4 Munich Cluster for Systems Neurology (SyNergy), D-80336 Munich, Germany; 5 Institute for Genetic and Biomedical Research, UoS of Milan, National Research Council, I-09042 Milan, Italy

**Keywords:** autophagy, atherosclerosis, endothelial cells, vascular smooth-muscle cells, immune cells, microRNAs

## Abstract

Atherosclerosis is a chronic inflammatory disease of the arterial vessel wall and underlies the development of cardiovascular diseases, such as myocardial infarction and ischemic stroke. As such, atherosclerosis stands as the leading cause of death and disability worldwide and intensive scientific efforts are made to investigate its complex pathophysiology, which involves the deregulation of crucial intracellular pathways and intricate interactions between diverse cell types. A growing body of evidence, including in vitro and in vivo studies involving cell-specific deletion of autophagy-related genes (ATGs), has unveiled the mechanistic relevance of cell-specific (endothelial, smooth-muscle, and myeloid cells) defective autophagy in the processes of atherogenesis. In this review, we underscore the recent insights on autophagy’s cell-type-dependent role in atherosclerosis development and progression, featuring the relevance of canonical catabolic functions and emerging noncanonical mechanisms, and highlighting the potential therapeutic implications for prevention and treatment of atherosclerosis and its complications.

## 1. Introduction

Cardiovascular diseases are the leading cause of death and disability worldwide and resulted in approximately 17.9 million deaths in 2019 [1,2]. Atherosclerosis is the chief cause of cardiovascular diseases and underlies the development of acute and chronic coronary syndromes as well as stroke and peripheral vasculopathy. Although initially viewed as a passive lipid accumulation into the arterial wall, solid evidence highlighted the relevance of immune and inflammatory responses [3,4,5]. Nowadays, we appreciate atherosclerosis as a non-resolving chronic inflammatory disease of the intima of large and medium-sized arteries in response to atherogenic lipoproteins [3,6].

Atherosclerosis is characterized by the build-up of fatty and fibrous tissue (i.e., atheroma) in the subendothelial space, which develops at predilection sites where perturbation of the laminar shear stress occurs. It begins as an impairment of the protective endothelial function triggered by multiple cardiovascular risk factors (e.g., dyslipidemia, hypertension, diabetes, smoking) which facilitate the accumulation of plasma lipoproteins (especially low-density lipoproteins, LDL) into the subendothelial space. The binding with negatively charged extracellular matrix proteoglycans favors the retention of LDL in the intima, where reactive oxygen species (ROS) and/or enzymes released by inflammatory cells mediate their oxidative modifications [3,7]. Oxidized LDL (oxLDL) aggravates endothelial cell dysfunction and provokes the intimal recruitment of immune cells by the secretion of inflammatory cytokines (e.g., Interleukin [IL]-1, IL-6, Granulocyte-Macrophage Colony-Stimulating Factor, GM-CSF) and chemotactic chemokines (e.g., CCL1, CCL2), as well as by the overexpression of adhesion molecules (e.g., Platelet endothelial cell adhesion molecule-1, PECAM-1; Intracellular adhesion molecule-1, ICAM-1; Vascular cell adhesion molecule-1, VCAM-1) [8,9]. At the initial stages (i.e., fatty-steak), the continuous gathering of oxLDL promotes the recruitment of circulating monocytes and the migration of tissue-resident macrophages and vascular smooth-muscle cells (VSMCs). These cells engulf oxLDL through scavenger receptors and give rise to lipid-laden foam cells, the hallmark of atherosclerosis [3,10,11]. During the progression of atherosclerosis, intraplaque cell death occurs by multiple mechanisms (i.e., necrosis, apoptosis, pyroptosis, and necroptosis) and eventually overwhelms the efferocytosis capabilities [12]. Thus, cell debris and cholesterol crystals accumulate within the plaque to make up the necrotic core, which typifies advanced lesions. As the lesion progresses to fibroatheroma, VSMCs, collagen, and other extracellular matrix molecules establish the fibrous cap, which covers the whole lesion and the necrotic core. The ‘shoulder’ regions of the fibrous cap are strongly infiltrated by T-cells, macrophages, and mast cells, which produce proteolytic enzymes and inflammatory mediators and contribute to the adventitial inflammation in advanced atherosclerotic plaques [3,6].

The clinical course of atherosclerosis is defined by an initial asymptomatic phase, during which the atheroma develops into the vessel wall of multiple arterial beds in proximity to bifurcations. This phase may last several decades and produces a slow, yet inexorable, narrowing of the arterial lumen that is initially counterbalanced by the physiological vasodilative ability of arteries and by collateral vascularization. When symptoms finally arise, they depend either on a reduction of blood flow caused by a stenosis which overcomes the reserve dilation capacities of the arteries, or by acute thrombotic obstruction triggered by plaque disruption or, more frequently, erosion [3,6,13,14]. Therapeutic management of atherosclerosis depends on the clinical phase. Whereas symptomatic acute events are subjected to strategies aimed at restoring blood flow (i.e., percutaneous coronary intervention or surgical endarterectomy), the chronic asymptomatic phase benefits from preventive measures [15]. Prevention of atherosclerosis involves the management of all modifiable cardiovascular risk factors to prevent—or even reverse—the progression of the disease. In an ideal world, these measures should start in early childhood by promoting a healthy lifestyle (i.e., primordial prevention). While lifestyle modifications definitely suit all individuals, pharmacological approaches are indicated for patients in high-risk groups for developing cardiovascular diseases. Effective approaches include lipid-lowering medications (e.g., statins, ezetimibe, Proprotein convertase subtilisin/kexin type 9 [PCSK9]-inhibitors), antiplatelet drugs (with a risk-to-benefit balance more favorable in secondary prevention), and proper management of hypertension, diabetes, and other metabolic disturbances [15,16,17,18,19,20,21,22]. Despite the current preventive approach, a substantial residual risk of developing cardiovascular events exists and represents an unmet need for cardiovascular research [23,24,25,26]. Recent randomized clinical trials (e.g., CANTOS, COLCOT) highlight the benefits of targeting inflammation for prevention of cardiovascular events [27,28,29], yet full comprehension of the mechanisms would hopefully allow the identification of further therapeutic targets to ultimately improve clinical management of atherosclerosis.

The pathophysiology of atherosclerosis encompasses intense crosstalk between multiple cell types (i.e., endothelial cells, immune cells, VSMCs) and the dysregulation of biochemical pathways crucial for cellular homeostasis, metabolism, and viability. Among them, autophagy has emerged as an essential process for physiological cell function whilst also playing a vital role in a broad range of diseases [30]. The essential role for autophagy in vascular function has been well established, and a first hint for its contribution in atherosclerosis came from the detection of autophagosome-like structures in human atherosclerotic plaques [31,32]. Here, we discuss the current evidence on cell-specific contributions of autophagy in the initiation, progression, and regression of atherosclerosis.

## 2. Generalities on Autophagy

Autophagy is amongst the most important catabolic processes carried out by eukaryotic cells to maintain nutrient homeostasis, promote adaptation to metabolic stress, and maintain genomic stability [33]. Classically thought of as a process for bulk recycling of intracellular components, autophagy now emerges as a coordinated adaptive pathway aimed at preserving cell viability and integrity in response to different stressors, including hypoxia, starvation, organelle dysfunction, metabolic anomalies, protein aggregation, and infections [34,35,36,37,38,39]. Initially discovered in yeast, it is mediated by a cluster of 16–20 evolutionarily conserved genes, named AuTophagy-related Genes (ATGs). There are three classes of autophagy, macro-autophagy, micro-autophagy and chaperone-mediated autophagy, which differ primarily through the method of cargo delivery to the lysosome for degradation. The mechanisms of micro-autophagy (where cytoplasmic components are directly engulfed in lysosomes) and chaperone-mediated autophagy (which involves chaperones to transport proteins and/or nucleic acids through the lysosomal membrane) have been extensively reviewed elsewhere [40,41,42]. This review will focus on macro-autophagy, commonly referred to as ‘autophagy’ [30].

The cellular hallmark of autophagy is the presence of double-membraned structures, named autophagosomes, originating as phagophores at specific sites on the endoplasmic reticulum known as omegasomes [43]. While engulfment of cargoes is classically viewed as a random mechanism, the discovery of selective autophagy shed a light on further roles of autophagy in several processes such as ageing, immunity, cancer, atherosclerosis, and cardiovascular diseases [30,44]. The mechanisms ruling cargo selectivity are under active investigation and involve the binding of components of the core autophagy machinery (e.g., LC3) to a cargo receptor, such as the proteins SQSTM1 (also known as p62) and Neurabin-1, NRB1 [44]. Peculiar examples of selective autophagy include: lipophagy (selective degradation of lipid droplets), mitophagy (selective degradation of dysfunctional mitochondria), pexophagy (selective degradation of peroxisomes), xenophagy (selective degradation of pathogens), reticulophagy (selective remodeling of the endoplasmic reticulum), and aggrephagy (selective degradation of aggregation-prone misfolded proteins) [44]. Both bulk and selective autophagy occur through six consecutive stages, namely: (1) induction and nucleation, (2) phagophore expansion, (3) cargo sequestration, (4) membrane sealing, (5) autophagosome maturation, and (6) fusion with a lysosome. Each of these steps is mediated by specific ATGs proteins and the current understanding of the molecular mechanisms have been reviewed elsewhere [33,45,46,47]. Here, we discuss selected aspects relevant to vascular physiology and atherosclerosis.

The upstream pathway of autophagy has been broadly studied and primarily involves the inhibition of the mechanistic target of rapamycin complex-1 (mTORC1), a master regulator of the metabolic activities of the cell [48]. The mTORC1 includes the serine/threonine kinase mTOR which phosphorylates and negatively regulates several proteins vital to the autophagy induction and nucleation. In basal condition, mTORC1 binds and inactivates Unc-51 like Autophagy Activating Kinase-1 (ULK1), ATG13 (both directly involved autophagy initiation), and represses Transcription factor EB (TFEB), a transcription factor governing autophagic and lysosomal gene expression [49]. However, autophagy can also be induced through the AMP-activated protein kinase (AMPK) pathway by direct activation of ULK1, VPS34, and Beclin-1 [50]. A wide range of stimuli has been shown to inhibit mTOR activity and promote autophagy, including nutrient deprivation, hypoxia, shear stress, and several drivers of atherogenesis (e.g., oxLDL, ROS) [31,33,50].

Besides the catalytic function, the autophagy machinery also mediates a range of noncanonical functions [51,52]. Indeed, given its multifaceted role in cellular homeostasis, autophagy lies at an interface with many cellular pathways, including cell death, cell cycle, and immune signaling [33,53]. Several ATGs participate in a phagocytic process similar to ‘classical’ autophagy, named LC3-associated phagocytosis, which involves selective degradation of extracellular components and utilizes only single-membrane-bound vesicles and a subset of ATGs [53]. Moreover, the autophagy machinery contributes to an unconventional secretory pathway, which allows for the extracellular secretion of proteins lacking an endoplasmic reticulum (ER)-targeting leader sequence. For example, ATG5 is required for the secretion of leaderless proteins such as IL-1β and IL-18 [54]. The autophagy pathway’s noncanonical function also plays a pivotal role in microRNA (miRNA) biogenesis and function. Indeed, autophagy regulates miRNA homeostasis through targeted degradation of miRNA-free DICER and AGO2, the main miRNA processing and effector proteins [55]. Furthermore, recent evidence suggests that the autophagy machinery contributes to the intracellular trafficking and to the secretion into extracellular vesicles of RNA-binding proteins and miRNAs [52,56].

## 3. Role of Autophagy in Different Cell Types

**Figure 1:**
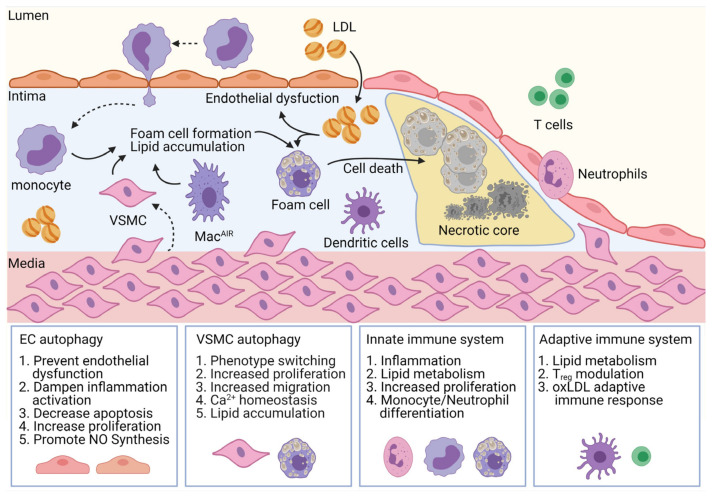
Cell-specific contribution of autophagy in atherogenesis. Autophagy governs homeostasis of cell types involved in all stages of atherogenesis. In endothelial cells (ECs), autophagy enhances nitric oxide (NO) bioavailability and prevents endothelial dysfunction and inflammatory activation, for example by lowering expression of adhesion molecules such as ICAM-1, VCAM-1, and PECAM-1. Autophagy regulates proliferation and migration of vascular smooth-muscle cells (VSMCs), contributes to their phenotypic switch, and regulates their lipid metabolism. Finally, autophagy machinery is involved in immune cell differentiation, proliferation, and lipid metabolism. Moreover, by affecting the adaptive immune system, it also contributes to the immune response to oxLDL epitopes and differentiation of T-cell subsets. LDL, low-density lipoproteins; Mac^AIR^, aortic intimal resident macrophages.

Clues of autophagy activation are detectable in all major cell types involved in the development of atherosclerosis [31,32]. The availability of transgenic mice models for inducing cell-specific deficiency of autophagy provided support for analyzing the role of autophagy in atherosclerosis and revealed its important, yet context- and cell-specific role. The following paragraphs summarize the mechanistic contribution of autophagy in distinct cell types during the development and progression of atherosclerosis (Figure 1).

### 3.1. Endothelial Cells

Endothelial cells make up the innermost layer of vessels known as the endothelium. This heterogeneous population of cells forms an important biological barrier with direct exposure to vessel contents throughout the whole vascular tree. Despite being only a thin monolayer, the endothelium is directly involved in sensing and responding to a multitude of bloodborne signaling molecules and hemodynamic changes [57,58]. Hence, it functions not just as a passive semipermeable barrier for vessel constituents, but rather as a vital regulator of vasodilation, angiogenesis, inflammation, and vessel homeostasis [59].

The term ‘endothelial dysfunction’ refers to a disorder in which the endothelium loses the physiological protective properties and associates with common cardiovascular risk factors (e.g., hypertension, dyslipidemia, diabetes) and stressors [60,61,62,63,64,65]. It encompasses a range of physiological changes to the vascular endothelium and represents a key early step in the development and progression of atherosclerosis [60,66]. Major hallmarks of endothelial dysfunction are the lower bioavailability of vasodilators such as nitrous oxide (NO) and the increase of endothelium-derived contracting factors, along with a pro-inflammatory proliferative and prothrombotic phenotype. This contributes to atherogenesis in the settings of chronic inflammation and hyperlipidemia [59,67].

**Figure 2:**
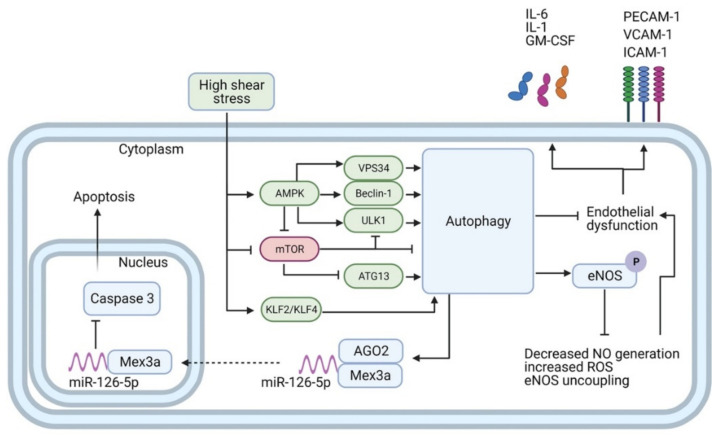
Autophagy transduces the effects of high-shear stress (HSS) in endothelial cells. HSS promotes autophagy through the activation of AMPK, KLF2, and KLF4, along with the suppression of mTORC1. AMPK in turn directly activates VPS34, Beclin-1, and UKL1, and represses mTORC1. mTORC1 is a repressor of autophagy, which when active binds to and inactivates both ULK1 and ATG13. Autophagy prevents endothelial dysfunction and dampens inflammation by reducing expression of inflammatory mediators (e.g., IL-1, IL-6, GM-CSF) and adhesion molecules (e.g., PECAM-1, ICAM-1, VCAM-1). Moreover, autophagy boosts NO biosynthesis by enhancing phosphorylation of eNOS and blocks eNOS uncoupling and ROS generation. Finally, autophagy mediates anti-apoptotic features by fostering the interaction and nuclear shuttling of Mex3a and miR-126-5p. This allows for the aptamer-like inhibition of caspase-3 function in the nucleus and reduction of apoptosis.

Autophagy plays a crucial role in maintaining homeostasis of the endothelium by regulating barrier integrity, vessel dilation, leukocyte adhesion, platelet aggregation, and angiogenesis. In terminally differentiated endothelial cells, such as those lining the blood vessels, autophagy allows for a high turnover of cytoplasmic contents imperative for cellular health and homeostasis, and it contributes to the adaptive response to laminar flow and physiological shear stress [68,69,70]. Compelling evidence from in vitro and in vivo studies have definitively proven that laminar shear stress promotes activation of the autophagic flux in endothelial cells (Figure 2), while endothelium in atheroprone areas exposed to low or perturbed shear stress (such as the inner curvature of the aortic arch) exhibits defective autophagy [56,70,71,72,73]. The upstream mechanisms of autophagy activation upon high shear stress are under intense investigation and involve the transcription factors Krüppel like factor (KLF)2 and KLF4, both strongly upregulated by high shear stress [56,71,74], as well as the activation of Fox01 by Sirt-1 [73]. On the other hand, low shear stress determines the inhibition of AMPKα and the activation of mTORC1, which culminate in the blockade of the autophagic flux [70,75]. All in all, evidence indicates that both low and high shear stress could induce the autophagic pathway, as detected by LC3-lipidation and the increase of Beclin-1 and Atg1. However, only laminar high shear stress promotes efficient autophagy, culminating in the degradation of autophagic cargoes, whilst the impaired autophagic flux observed in low shear stress areas contributes to cell death, senescence, inflammation, and favors atherosclerotic development [70,72,75,76].

Overall, the protective role of endothelial autophagy against atherosclerosis is undoubtedly supported by the evidence that endothelial-specific deletion of the *Atg5* and *Atg7* genes in mice with an atheroprone background (*Apoe^−/−^*) increases atherosclerotic burdens upon exposure to chronic hyperlipidemia [56,70,77]. The mechanisms beyond the atheroprotective effect have been deeply investigated, and the links between endothelial autophagy and NO bioavailability, ROS generation, inflammation, and apoptosis have been well established. In particular, endothelial cells synthesize NO starting from the amino acid L-arginine by means of the enzyme endothelial NO synthase (eNOS), which localizes on the Golgi apparatus or in the *caveolae*. Although constitutively expressed, the transcription of eNOS is strongly enhanced by exposure of endothelial cells to laminar shear stress. Moreover, the activity of eNOS is also regulated by the phosphorylation of a serine residue (p-eNOS^S1177^, in the human homologue) [78,79]. The contribution of autophagy in these processes has been detailed and involves the signaling pathway of the P2Y1 purinergic receptor and the protein kinase C-δ (PKCδ) [72]. Indeed, disruption of the autophagic machinery by genetic silencing of *Atg3* prevents phosphorylation of eNOS at its positive regulatory site S1117 (p-eNOS^S1177^) and dampens the activation of eNOS, thus reducing NO generation and endothelial-dependent vessel dilatation [72].

Besides its role in the production of NO, upon exposure to low shear stress or under pathological conditions (e.g., diabetes, hypercholesterolemia), eNOS contributes to the reduction of molecular oxygen (O_2_) to generate superoxide anion (O_2_^−^), which preludes the formation of most ROS, in a process known as ‘eNOS uncoupling’ [80]. In this process, the reduction of molecular oxygen is no longer coupled to oxidation of L-arginine, and the electrons leak from the reductase domain of eNOS during their transport and are transferred to oxygen molecules forming super-oxides rather than NO [80,81]. Moreover, super-oxides may interact with NO, leading to the production of peroxynitrite, a strong oxidant agent which boosts oxidative damage [82]. The consequent build-up of ROS leads to a local pro-inflammatory response [69,83]. A variety of mechanisms underlie the eNOS uncoupling, including the deficiency or the oxidation of tetrahydrobiopterin, an essential eNOS cofactor, lack of L-arginine, and posttranslational modification of eNOS, such as S-glutathionylation of cysteine residues in the reductase domain or phosphorylation of negative regulatory residues [80,84,85]. A protective role of endothelial autophagy in ROS generation has been proven in endothelial cells exposed to atheroprone low shear stress in vitro (2 dyne/cm^2^) or in the carotid ligation model in vivo. Both conditions resulted in an impairment of the autophagic flux and eNOS uncoupling. Interestingly, add-on of mTORC-inhibitors (rapamycin or WYE-354) or overexpression of *Atg5* to enhance the autophagic flux reduced the accumulation of ROS, proving the importance of effective autophagy in preventing eNOS uncoupling [76]. The molecular mechanisms underlying these processes have been investigated and involve the phosphorylation of a threonine residue (p-eNOS^T495^, in the human homologue) which is dampened by induction of autophagy, which rather promoted phosphorylation of the positive regulatory site p-eNOS^S1177^ [72,76]. Moreover, accumulation of dysfunctional organelles (i.e., mitochondria) also contribute to the exaggerated ROS production observed upon genetic silencing of *Atg3* and *Atg5* in endothelial cells [72].

Breaches in the endothelial integrity allows for the deposition of modified lipoproteins (e.g., oxLDL), favors the infiltration of leukocytes during atherosclerosis development, and exposes the underlying thrombogenic collagen in atherosclerosis complications [86,87]. Autophagy acts as a cytoprotective mechanism to prevent cell senescence and death in arterial tracts exposed to high laminar shear stress [56,70]. Conversely, endothelial cells exposed to low shear stress or deficiency in endothelial autophagy exhibit higher apoptosis rates and are unable to align in the direction of the blood flow, a hallmark of endothelial cell health [70,75]. A classical mechanism mediating the inhibitory effect on apoptosis is the ability of autophagy to sequester and degrade dysfunctional mitochondria. Indeed, damaged mitochondria release catabolic hydrolases and caspase activators (e.g., cytochrome *c*) into the cytoplasm and dissipate the inner mitochondrial transmembrane potential, thus producing an ‘energetic catastrophe’ that represents an irreversible commitment to cell death [88,89].

It is noteworthy that non-degradative functions of autophagy also play a crucial role in protecting endothelial cells from apoptosis. In endothelial cells exposed to high shear stress or treated with the mTOR-inhibitor rapamycin, the autophagy machinery promotes a specific interaction between the RNA-binding protein Mex3a and miR-126-5p, a miRNA highly enriched in endothelial cells [56,90,91]. This interaction occurs on the extraluminal surface of autophagosomes, involves the argonaute protein AGO2, and eventually results in the nuclear translocation of miR-126-5p. Inhibition of autophagy through chemicals (e.g., 3-methyladenine) or genetic (i.e., silencing of ATG5 or ATG7) approaches impedes the nuclear shuttling of miR-126-5p [56]. Once in the nucleus, miR-126-5p dissociates from AGO2 and engages into aptamer-like interactions with the effector caspase-3, thus impeding proper assembly of its catalytic active site, and ultimately dampening apoptosis to preserve endothelial cell viability [56]. The direct inhibition of protein activity by miR-126-5p outlines a novel noncanonical mechanism of miRNA action that contributes to the autophagy–apoptosis crosstalk.

Likewise, autophagy plays an essential role in endothelial response to other atherogenic stimuli. In particular, it exerts a crucial role in limiting lipid accumulation within vessel walls and protecting against vascular stressors, including oxidative stress, high glucose, and oxLDL [69]. Exposure to oxLDL activates autophagy in endothelial cells and stimulates sequestration of the lipoprotein excess into autophagosomes [77,92]. Consistently, endothelial-specific *Atg7* deletion in *Apoe^−/−^* mice resulted in the increased accumulation of oxLDL in the arterial wall, proving the impact of endothelial autophagy in preventing exogenous lipid accumulation within the vessel wall [77]. However, depending on the concentration, treatment with oxLDL promotes both apoptosis and autophagy to participate in an inhibitory crosstalk with autophagy, preventing progression of apoptosis for lower doses of oxLDL, while apoptosis overwhelms autophagy for higher concentrations [92,93]. Finally, inhibition of autophagy has been shown to worsen endothelial damage caused by high-glucose and excessive Angiotensin II stimulation, as well as to dampen the protective effect of Angiotensin-converting enzyme (ACE)-inhibitors and angiotensin receptor blockers on endothelial cell viability [89].

The involvement of the vascular endothelium in the pro-inflammatory environment that drives atherosclerosis is well established. Preceding initiation of atherosclerotic plaques, endothelial cells undergo a dramatic change in phenotype. This is thought to be driven by the pleiotropic activation of NF-κB, resulting in the expression of a range of pro-inflammatory proteins in the intact endothelium overlying atherosclerotic plaques and precedes the earliest recruitment of mononuclear leukocytes to the developing lesion [3,67]. Autophagy has been shown to play an important role in the immune response [94], and to antagonize age-related vascular inflammation [95]. Whilst further investigation is required to fully elucidate the role autophagy in the pro-inflammatory endothelial phenotype, deficiency in endothelial autophagy (*Atg5* silencing) aggravates endothelial inflammation in response to the pro-inflammatory stimulus TNF-α, as proven by higher ICAM-1 expression and higher CCL2 release [70]. Furthermore, the anti-inflammatory effects of miR-100 in endothelial cells are mediated by repression of several components of the mTORC1 pathway, which is itself a repressor of autophagy [96].

In all, endothelial autophagy plays an important role in the physiology and pathophysiology of both the vascular endothelium and atherosclerosis. By mediation of key physiological properties, such as barrier integrity, vessel dilation, leukocyte adhesion, platelet aggregation, and angiogenesis, autophagy maintains vascular homeostasis. Furthermore, during the development and progression of atherosclerosis, it also has an important role in responding to ROS and lipid accumulation, apoptosis, and the promotion and resolution of both acute and chronic inflammation. Whilst the field of endothelial autophagy is rapidly expanding, further investigation into the clinical efficacy and new modalities for targeting specific EC autophagy are needed.

### 3.2. Vascular Smooth-Muscle Cells (VSMC)

Vascular smooth-muscle cells (VSMC) are the most abundant cell-type of the arterial vessel wall. In healthy arteries, terminally differentiated VSMCs reside in the media and respond to changing hemodynamic conditions through elastic recoil, extracellular matrix (ECM) production, and regulating the arterial tone and diameter [97]. They also significantly contribute to other physiological processes such as tissue homeostasis and repair, as well as in pathological processes, including atherosclerosis [11,98]. Even in a healthy vessel, fully functional and fully differentiated VSMCs retain remarkable phenotypic plasticity. Indeed, VSMCs can undergo phenotypic modulation in response to injuries, resulting in what is commonly referred to as a synthetic phenotype. The shift from a contractile to a synthetic phenotype promotes migration of VSMCs toward the intima during atherogenesis, increases the proliferative potential, raises the expression of scavenger receptors, and enhances the secretion of ECM components and proteases [99]. These changes are pivotal for the stability of atheromas, thus dictating the fate of atherosclerotic lesions [97]. Moreover, VSMCs considerably contribute to the population of foam cells, as they can account for up to 50% of foam cells in human atherosclerotic plaques [100].

Early ultrastructural studies by electron microscopy revealed the presence of autophagosomes in atherosclerotic plaques, thus corroborating the activation of autophagy in VSMCs in vivo in human and animal models of atherosclerosis [32,101,102]. Various atherogenic stimuli can promote autophagy in VSMCs, including lipid species, cytokines (e.g., TNF-α, CD40-CD40L), growth factors (e.g., Platelet-derived growth factor, PDGF; insulin-like growth factor 1, IGF-1), and metabolic stressors [103]. For example, exposure to moderate doses of oxLDL (10–40 µg/mL) promotes autophagy in VSMCs in vitro through a pathway involving the scavenger receptor LOX1 and its regulating miRNA, let-7g [92]. Interestingly, autophagy was mitigated by higher oxLDL concentrations which rather elicited apoptosis [92]. The inhibition of the protective mitophagy observed for higher concentrations of oxLDL contributes to this negative crosstalk, impeding efficient removal of dysfunctional mitochondria [104]. The relevance of functional autophagy in the maintenance of a mitochondrial population in VSMCs has also been proven in vivo. Indeed, VSMC-specific impairment of the autophagy machinery (obtained in *Tagln^Cre+^Atg7^flox/flox^* mice) leads to accumulation of fragmented mitochondria with reduced bioenergetic efficiency, increased oxidative stress, and enhanced apoptosis. This takes part in the progression of atherosclerosis toward a vulnerable phenotype characterized by large necrotic core formation and thinner fibrous caps [105]. Indeed, VSMCs form the principal constituent of the atheroma’s fibrous cap and contribute to its thickening by producing ECM components [105]. As a thick fibrous cap provides stability to an atherosclerotic plaque, autophagy in VSMC is a critical determinant of arterial pathology, preserving VSMC viability and protecting them against apoptotic cell death [88,105,106]. Altogether, these results strengthen the intense crosstalk between autophagy and apoptosis in atherogenesis. Whereas they can be elicited by common upstream stimuli (e.g., oxLDL), they divergently affect cell viability and ultimately, disease progression.

Besides being an essential mechanism for survival, autophagy is also a critical regulator of the VSMCs phenotype and function. In particular, it contributes to Ca^2+^ homeostasis and contractile capacity of VSMCs. Defective autophagy in VSMCs results in higher basal Ca^2+^ concentrations and enhanced vascular reactivity [107]. Furthermore, autophagy is a crucial regulator of VSMC phenotype and function along with the cellular stress response. The remarkable VSMC plasticity in atherosclerosis has been confirmed using lineage-tracing experiments and majority of VSMCs in atherosclerotic plaques display altered phenotypes, such as proliferative and synthetic or macrophage-like [11,106,108,109,110,111]. Evidence supports the relevance of autophagy in the proliferative and synthetic response of VSMCs to stimuli, as well as in migration. In particular, the platelet-derived growth factor (PDGF) is a primary modulator of VSMCs phenotypic switch, and pharmacological inhibition of PDGF signaling dampens VSMC proliferation, migration, and neointima formation [112]. Autophagy is rapidly activated by PDGF through an mTOR-independent pathway and contributes to the phenotypic switch by reducing the expression of contractile proteins (e.g., α-sm-actin), increasing synthetic VSMC markers (e.g., vimentin), and enhancing migration and proliferation in vitro [97]. Conversely, pharmacological inhibition of autophagy using different inhibitors (i.e., 3-methyladenine, spautin-1, bafilomycin A1) stabilized the VSMC contractile phenotype in vitro, decreasing the degradation of α-sm-actin, preventing proliferation and migration, and dampening synthesis of collagen upon treatment with PDGF [97]. Moreover, the VSMC-enriched miR-145 has been shown to inhibit proliferation and migration of VSMCs by suppressing autophagy [113]. Finally, defective autophagy determined the loss of the classical spindle-shaped phenotype of VMSC in favor of bigger cells with rhomboid shape with enhanced migration as a result of upregulation of Matrix metallopeptidase (MMP)- 9, transforming growth factor (TGF)-β, and CXCL12 [106], all of them involved in atherosclerosis [114,115,116].

Finally, recent evidence supports a role of autophagy in VSMCs differentiation into foam cells. Indeed, the P2RY12 receptor inhibits autophagy in VSMCs via the PI3K-Akt-mTOR pathway, thus decreasing cholesterol lipolysis and promoting conversion to foam cells in vivo [117]. Moreover, investigations on the role of the sterol regulatory element binding protein (SCAP) showed that it can induce autophagy to decrease oxidative stress and lipid accumulation [118]. In this context, SCAP acts as a cholesterol sensor and maintains intracellular cholesterol levels by regulating uptake and de novo cholesterol synthesis. This pathway is altered in *Apoe^−/−^* mice with conditional deletion of *Scap* in VSMCs (*Apoe^−/−^Tagln^Cre+^Scap^flox/flox^* mice), showing enhanced autophagy and reduced aortic plaque burden compared to controls [118]. Altogether, these data suggest the multifaceted protective action of VSMCs autophagy in the maintenance of arterial homeostasis and in prevention of atherosclerosis development toward a high-risk vulnerable phenotype.

### 3.3. Innate Immune System

The innate immune system is deeply involved in the pathogenesis of atherosclerosis, and arterial macrophages play important roles in promoting and resolving inflammation, vasculature repair, and regeneration [119]. Macrophages are the most abundant myeloid cells in atherosclerotic plaques and participate in atherosclerosis progression by cholesterol lading, clearance of cellular debris, cholesterol efflux, production of inflammatory mediators, and proteolytic enzymes that promote plaque destabilization [3,6]. There are two main sources of macrophages in atherosclerosis: (i) arterial-resident macrophages that differentiate into the first foam cells within the arterial intima, and (ii) monocyte-derived macrophages which are recruited from blood and migrate into the subendothelial space in response to inflammatory stimuli [120,121,122]. Both tissue-resident and monocyte-derived macrophages show high plasticity and adapt to different stressors by acquiring distinct functional phenotypes [123]. Although macrophages may initially protect against oxLDL accrual, with the progression of the disease, their migration capacity and ability to catabolize exogenous cargoes and dysfunctional organelles are strongly reduced [124,125].

Autophagy crucially modulates immune response and the inflammasome. Aside from the clearing of dysfunctional lysosomes, it has a range of roles, including the antigen presentation, the removal of inflammasome activating signals, and the degradation of inflammasome components [126]. Significant dysfunction of autophagy in lesional macrophages develops during the progression of atherosclerosis, as shown by the accumulation of the autophagic substrate p62 in macrophages in human and murine atheromas [124,127,128]. Moreover, the expression of the autophagy-related protein ATG16L1 was also detected in macrophage-rich areas of advanced human carotid atherosclerotic plaques [129]. Mechanistic studies in vivo have shown that disruption of autophagy in macrophages (in LysM^Cre+/^^−^Atg5^flox/flox^ mice) leads to a marked increase in atherosclerosis burden [127,130]. In detail, inhibition of autophagy worsens NADPH-dependent oxidative stress and promotes the development of plaques with larger necrotic cores due to the higher apoptosis and impaired phagocytosis and efferocytosis [130]. Moreover, dysfunctional autophagy activates NLPR3 inflammasome in macrophages and increases the secretion of IL-1β [127]. Interestingly, no effect on atherosclerosis was observed in mice with haploinsufficiency of autophagy (i.e., Apoe^−/−^Becn1^−/+^), with an only partial defect in the autophagy machinery, thus suggesting that promotion of atherosclerosis requires dramatic disruption of basal autophagy [127].

Lipoprotein uptake by macrophages is an early pathogenic event in atherogenesis. Several mechanisms have been identified by which macrophage clear the lipids and debris in the atherosclerotic plaque [131,132], and autophagy contributes to lipid clearance and cholesterol efflux from macrophages [133]. Indeed, macrophages use lipophagy, a specific form of selective autophagy, to deal with the lipid accumulation and regulation of cholesterol efflux [133,134]. In early atherosclerosis, foam cells mainly accumulate lipids into cytoplasmic lipid droplets, while macrophages in advanced plaques show preferential engulfment into lysosomes. In cholesterol-laden macrophages, autophagy is induced with lipid loading. It actively mobilizes cholesteryl esters from lipid droplets to lysosomes, where it is hydrolyzed through the lysosomal acid lipase pathway to generate free cholesterol, which is a suitable substrate for ATP-binding cassette transporter (ABCA1)-mediated cholesterol efflux [133]. Consistently with this pathway, silencing of Atg5 diminished the cholesterol efflux to ApoA-I (ABCA1-dependent) in vitro and dampened the reverse cholesterol transport in vivo [133]. The upstream pathways regulating autophagy activation in lesional macrophages have been investigated and involve the activation of the transcription factor TFEB, which coordinates a broad network of genes related to autophagy and lysosomal pathways [124]. Overexpression of TFEB decreased lipid-induced macrophage apoptosis and IL-1β production in vitro, while reducing the development and promoting a more stable phenotype of atherosclerotic plaques in vivo [124].

Finally, neutrophils also play a role in the mechanisms of atherosclerosis. Indeed, hyperlipidemia induces neutrophilia in Apoe^−/−^ mice and neutrophil depletion at the early stages reduces atherosclerosis and affects plaque infiltration of monocyte/macrophages, in line with the ability of neutrophils to recruit monocytes and dictate their fate [135,136,137,138]. Moreover, the release of protein contained in neutrophil granules or the production of neutrophil extracellular traps (NETs) contributes to plaque progression and destabilization [139]. Besides its role in early granulopoiesis [140,141], autophagy is involved in the regulation of neutrophil degranulation and deletion of Atg5 or Atg7 inhibits the release of granule-derived mediators and the production of ROS in vitro and in vivo [142]. Although evidence suggests that autophagy may be associated with NETosis (i.e., NET-mediated cell death), the detailed molecular mechanisms are not completely cleared [143,144,145,146]. Indeed, neutrophils with lower expression of Atg5 (e.g., in aged mice) display reduced release of NETs [143]. In contrast, genetic deletion of Atg5 or inhibition of autophagy at late stages (by bafilomycin A1) did not affect the ability of neutrophil to produce NETs [144]. Moreover, a study on arterial thrombosis has shown that mTORC1 inhibition and autophagy regulate NETosis triggered by the inorganic polyphosphate released by activated platelets during myocardial infarction [145]. Similarly, rapamycin facilitated NET-release from human neutrophils exposed to TNF-α and anti-neutrophil cytoplasmic (ANCA)-antibodies [146]. Despite the inconsistences, autophagy has been implicated in NETosis in human sepsis and in other inflammatory and autoimmune diseases [147,148,149,150], however studies specifically investigating the contribution of neutrophil autophagy in atherosclerosis are still lacking.

### 3.4. Adaptive Immune System

Besides the relevance of innate immunity, adaptive immune response contributes to atherosclerosis and a T-cell-mediated autoimmune component has been described [151]. The role of autophagy in T-cell biology has been established [152,153], however our understanding of the role of T-cell autophagy in atherosclerosis is still developing. Differences in T-cell subsets and dendritic cells have been observed in human vulnerable plaques and could impact mechanisms contributing to plaque stability and progression [154]. In general, CD4^+^ T-helper 1 (T_H1_) cells and natural killer T-cells are deemed of proatherogenic features [155]. In atherosclerotic plaques, the interaction of CD4^+^ T-cells with antigen-presenting cells (APCs) leads to the secretion of pro-inflammatory cytokines and the expression of surface markers (e.g., CD44), indicating that they have been exposed to cognate antigens (e.g., ApoB) and armed for a rapid response [156]. This involves the secretion of IL-2, IFN-γ, and TNFα, and associates with plaque instability [157,158,159,160,161,162]. On the contrary, T_reg_ cells appear atheroprotective in mice and humans, as suggested by the inverse correlation detected between cytokines secreted by T_reg_ cells (e.g., IL-10) and cardiovascular diseases [158,159,160,161,162]. Controversy still exists on the role of other T-cell subsets (e.g., T_H2_, T_H17_, CD8^+^ T cells, γδ T-cells) in atherosclerosis progression as well as the relevance of the conversion of T_reg_ cells into pro-inflammatory T-cell subsets during atherogenesis [155].

Autophagy is crucial for the function of different subsets of T-cells and in different developmental stages [151]. The essential relevance of ATGs for the functional stability of both T_reg_ and T_H1_ cells has been clearly shown in studies involving conditional deletion of *Atg5* and/or *Atg7* [163,164,165,166]. The relevance of T-cell autophagy in the development of atherosclerosis has been investigated using T-cell-specific *Atg7* deletion (in *Lck^Cre+^Atg7^flox/flox^* mice) after adenoviral-mediated overexpression of *Pcsk9*. In this study, deficiency of T-cell autophagy resulted in a reduction of the burden of atherosclerosis without affecting macrophage infiltration, in lower CD4^+^ and CD8^+^ cells in peripheral lymphoid tissues and limited inflammatory potency in naïve T-cells [151].

Dendritic cells (DCs) are professional APCs found in atherosclerotic plaques [167]. The contribution of DCs in atherogenesis is exerted through their role in lipid metabolism and in modulation of T_reg_ responses and T-cell activation [168,169,170]. Albeit dispensable for DC development, autophagy contributes to DC function and affects a range of processes, including maturation, migration, cytokine production, migration, and T-cell activation [171]. The autophagic flux is activated in DCs during atherogenesis and disruption of autophagy in DCs (in *Cd11c^Cre+^Atg16l1^flox/flox^* mice) elicits an immunosuppressive response, promotes the expansion of T_reg_ cells, and limits atherosclerosis [170]. Given the relevance of autophagy in regulating functional properties of DCs, and their impact in innate and adaptive immune response, targeting autophagic pathways in specific DC subsets may provide insights into their plasticity and potential therapeutic chances in atherosclerosis.

Finally, macrophages also have an important role in mounting an adaptive immune response to oxLDL present in atherosclerosis. Not surprisingly, arterial resident-macrophages in the aortic arch were identified as dendritic cells due to the expression of specific antigen-presenting components (e.g., the major histocompatibility complex class II, MHC-II), while their macrophage nature has been highlighted by recent lineage-tracing models and transcriptional profiling [10]. Interestingly, autophagy can regulate the MHC-II-antigen presentation on the macrophage surface through the endosomal/lysosomal degradation of internalized antigens, including oxLDL [172]. Indeed, exposure to oxLDL promotes the activation of autophagy and exposition of MHC-II in a pathway that involves the protein spleen tyrosine kinase (SYK). In particular, in response to oxLDL exposure, the phosphorylation of SYK activates the MAPK8/9 pathway, resulting in the release of Beclin-1 from its complex with BCL2 and in autophagy induction [172]. Interestingly, this pathway is also confirmed in vivo, and hypercholesterolemic mice with genetic deletion of Syk show reduced surface expression of MHC-II in macrophages, and reduced antibody responses to oxidation-specific LDL epitopes [172].

## 4. Therapeutic Implications

As discussed above, solid data show a generally protective role of autophagy in atherosclerosis. Hence, the possibility to enhance the autophagic status of cells involved in atherosclerosis may assume clinical relevance as a potential preventive treatment for atherosclerosis. However, no drug is available to specifically target autophagy for clinical use in human diseases. A common approach to pharmacologically initiate autophagy is the inhibition of mTORC1 by using rapamycin (also known as sirolimus) or its derivatives (e.g., everolimus, zotarolimus). Preclinical studies on animal models of atherosclerosis have shown that systemic administration of rapamycin may reduce atherosclerosis burden and inflammatory status [173,174,175,176,177]. Rapamycin and derivatives find clinical application in drug-eluting stents deployed during percutaneous coronary interventions to prevent restenosis [178]. It is likely that other features of mTORC1 inhibition (e.g., inhibition of cell proliferation, blockage of protein and nucleotide synthesis, inhibition of metabolism) as well as the chronic inhibition of mTORC2 contribute to the effect of rapamycin [48,179]. It is noteworthy that everolimus-eluting stents placed in diseased arteries of cholesterol-fed rabbits promoted the clearance of intraplaque macrophages and was accompanied by evidence of autophagosome-like structures [180]. Nonetheless, given the relevance of the multiple mTORC-regulated pathways for cellular homeostasis, it is not surprising that rapamycin and mTORC inhibitors may produce side effects which are sometimes serious or debilitating [181].

For this reason, approaches to regulate autophagy independently on mTORC inhibition have been tested. Trehalose is a disaccharide found in several non-mammalian species which acts as a chaperone to prevent protein denaturation [182]. Although the detailed mechanism is still elusive, trehalose can enhance autophagy in mammalians via a mTORC-independent pathway. Interestingly, systemic administration of trehalose stimulated macrophage autophagy in vivo and decreased atherosclerosis in *Apoe^−/−^* mice, but not in mice with myeloid-specific deletion of *Atg5*, pinning the relevance of macrophage autophagy on the beneficial effects of trehalose on atheroprogression [124]. A beneficial cardiovascular effect of trehalose in humans is suggested by a small interventional study showing that administration of trehalose (100 g/day) enhanced microvascular function [183], and upcoming studies (e.g., IR-TREAT study, ID: NCT03700424) will contribute to our understanding on the therapeutic opportunity of trehalose. The polyamine spermidine also promotes autophagy, mainly by inhibiting the histone acetyltransferase p300. Administration of spermidine reduced lipid accumulation and decreased the size of the necrotic core in atherosclerotic plaques of *Apoe^−/−^* mice, but not in mice with VSMC-specific deletion of *Atg7* [184]. A translational perspective to these findings is provided by epidemiological evidence showing an inverse correlation between dietary intake of spermidine and incident cardiovascular diseases [185]. Moreover, low dietary intake of spermidine was associated with higher plasma levels of chitinase-3-like protein-1 (CHI3L1), which is involved in atherosclerotic plaque inflammation and disruption [185]. Finally, an established mTORC-independent autophagy inducer is the calcium-channel blocker verapamil. This drug is approved for treatment of cardiovascular diseases (e.g., hypertension, angina, paroxysmal supraventricular tachycardia), however it has also shown autophagy-dependent cytoprotective effects in VSMCs [186].

While autophagy-specific treatments for atherosclerosis will require further research, medications routinely used in cardiovascular medicine also affect autophagy. Albeit being the most prescribed lipid-lowering agent, statins also act via pleiotropic mechanisms that are independent on the lowering of plasma LDL [187,188]. The influence of statins on autophagy has been proven by in vitro and in vivo experiments. For example, atorvastatin enhances the autophagic flux in macrophages to prevent intracellular accumulation of oxLDL in vitro and its administration in *Apoe^−/−^* mice increases the expression of autophagy markers in atherosclerotic plaques and reduces plaque size and vulnerability [189]. The effect of statins on autophagy is, however, heterogeneous, and depends on the specific statin, the cell type, and baseline levels of autophagy [190]. A class of clinically approved anti-hypertensive drugs, the angiotensin-receptor blockers, has also been shown to promote autophagy. For example, telmisartan and irbesartan promote autophagy through the proliferator activated receptor-γ (PPARγ) and telmisartan attenuates lipid accumulation in VMSCs, which is rescued by Atg7 knockdown [191,192]. Finally, the widely used anti-diabetic drug metformin is an established inducer of endothelial cells and VSMCs autophagy and exerts anti-atherosclerotic features [193,194,195,196].

However, targeting autophagy for therapeutic purposes poses challenges to be overcome before clinical use. Indeed, autophagy plays a crucial homeostatic role in all eukaryotic cells and is involved in multiple diseases. Thus, the benefit-to-risk balance of systemic autophagy modulation should be wisely evaluated. For example, while evidence anticipates a possible beneficial outcome for autophagy enhancement in atherosclerosis, nonetheless, autophagy may confer a survival advantage to neoplastic cells and its inhibition may reduce the growth of advanced cancers [197]. Novel technologies for a theranostic-controlled release with stimuli-responsive potentials are under development and include the use of nanocarriers such as liposomes, carbon dots, and others [198]. Furthermore, autophagic flux is a highly dynamic process and its impairment may occur at different stages: it may defect in its induction (no formation of autophagosomes), in the interference with cargo recognition, or in the autophagosome–lysosome fusion (lack of autophagosome clearance), thus the best therapeutic target should be determined and is likely context- and cell-specific. As mentioned, the majority of available autophagy inducers act through mTORC inhibition, which is associated with severe adverse effects and tolerance upon chronic administration. Besides the abovementioned natural compounds (trehalose and spermidine), other drugs and additional targets (e.g., ATG7, ATG4B) are currently scrutinized for their ability to enhance autophagy [199,200]. Finally, although autophagy often acts as a cell-survival mechanism, exaggerated induction of autophagy may promote autophagic cell death [88], thus the therapeutic window for autophagy modulation should be properly determined and targeted.

## 5. Conclusions

Autophagy plays a crucial role in the cell types involved in atherosclerosis and its deficiency commonly results in worse outcomes in terms of atherosclerosis development and progression toward vulnerability. Further research is needed for a comprehensive understanding of its role in the intense intercellular crosstalk of atherogenesis and for the identification of the best drug candidates and delivery systems. Nevertheless, pharmacological modulation of autophagy undoubtedly appears as an attractive therapeutic opportunity to reduce the residual cardiovascular risk in patients with atherosclerosis and to ultimately reduce the burden of cardiovascular diseases.

## Data Availability

No new data were created or analyzed in this study. Data sharing is not applicable to this article.
